# Above and beyond state-of-the-art approaches to investigate sequence data: summary of methods and results from the population-based association group at the Genetic Analysis Workshop 19

**DOI:** 10.1186/s12863-015-0310-0

**Published:** 2016-02-03

**Authors:** Justo Lorenzo Bermejo

**Affiliations:** Statistical Genetics Group, Institute of Medical Biometry and Informatics, University of Heidelberg, Im Neuenheimer Feld 305, 69120 Heidelberg, Germany

## Abstract

This paper summarizes the contributions from the Population-Based Association group at the Genetic Analysis Workshop 19. It provides an overview of the new statistical approaches tried out by group members in order to take best advantage of population-based sequence data.

Although contributions were highly heterogeneous regarding the applied quality control criteria and the number of investigated variants, several technical issues were identified, leading to practical recommendations. Preliminary analyses revealed that Hurdle-negative binomial regression is a promising approach to investigate the distribution of allele counts instead of called genotypes from sequence data. Convergence problems, however, limited the use of this approach, creating a technical challenge shared by environment-stratified models used to investigate rare variant-environment interactions, as well as by rare variant haplotype analyses using well-established public software. Estimates of relatedness and population structure strongly depended on the allele frequency of selected variants for inference. Another practical recommendation was that dissenting probability values from standard and small-sample tests of a particular hypothesis may reflect a lack of validity of large-sample approximations. Novel statistical approaches that integrate evolutionary information showed some advantage to detect weak genetic signals, and Bayesian adjustment for confounding was able to efficiently estimate causal genetic effects. Haplotype association methods may constitute a valuable complement of collapsing approaches for sequence data. This paper reports on the experience of members of the Population-Based Association group with several novel, promising approaches to preprocessing and analyzing sequence data, and to following up identified association signals.

## Background

Every 2 years, participants of the Genetic Analysis Workshop (GAW) explore a common data set using novel approaches and summarize their findings in a short paper. Contributions to the GAW19, held August 24–27, 2014, in Vienna, Austria, were split up by workshop organizers into 9 thematic groups. The present article summarizes the methods and results from the Population-Based Association group, aiming at providing a motivating, intuitive overview of the new approaches tried out by group members. Technical details and descriptions of individual contributions can be found in the publications *BMC Proceedings* and *BMC Genetics*.

Members of the Population-Based Association group worked in pairs in the weeks preceding the GAW. Each participant contacted the other pair member, read the preliminary version of his/her individual contribution, and discussed findings and results with him/her. On the first day of the workshop, group members briefly presented the contributions of the other pair member. Engaged discussions and intensive team work during 4 group meetings and a poster session led to a consensus summary of the group contributions, which was presented to all GAW participants in a plenary session.

**Fig. 1:**
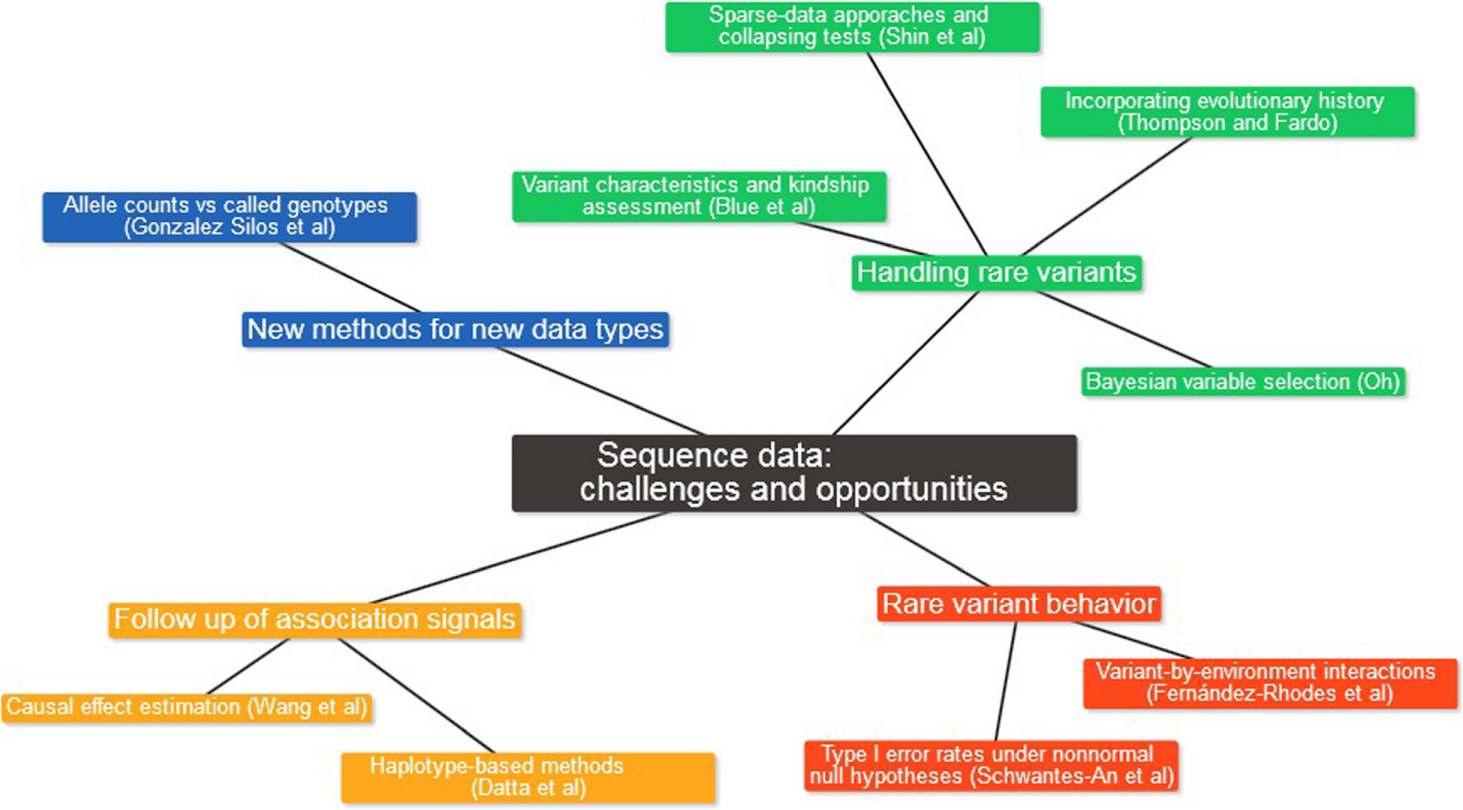
Mind-map with the 9 accepted contributions from the Population-Based Association group

After submission and peer review, 9 individual papers from the Population-Based Association group were accepted for publication in the GAW19 proceedings [1–9]. Figure 1 represents a mind-map of the 9 accepted contributions. Members of our group explored novel approaches to circumvent the limitations of current methods, and to take most advantage of next-generation sequence data. Individual contributions could be classified into 4 loose themes: development of new methods for new types of data; manipulation of rare variants; behavior of rare variants acting alone and interacting with environmental factors; and the follow up of association signals from sequence data.

## Methods

### Material

Table 1 summarizes the genotypes and phenotypes investigated by group members, and the applied filters for quality control. Although most group members analyzed whole exome sequence data in odd-numbered autosomes from unrelated individuals, some participants focused on genetic variability in the *MAP4* gene. Regarding investigated phenotypes, the use of real and simulated data was well-balanced. Two participants defined affected cases as individuals with a systolic blood pressure greater than 140 mm Hg, or a diastolic blood pressure greater than 90 mm Hg, or taking antihypertension medication. A group member simulated their own phenotypes. The applied quality control filters were highly heterogeneous. For example, the threshold for variant exclusion owing to missing calls varied from 5 to 25 %. Also the number of investigated variants showed a large variability. In contrast to a group member who considered 88 variants in 2 genes, another participant examined more than 313,340 variants in odd-numbered autosomes.

**Table 1:** Genotypes, phenotypes, and quality control filters applied by authors of accepted papers in the Population-Based Association group

Contribution	Genotypes	Phenotypes	Quality control
Blue et al. [2]	GWSNPA data for odd-numbered autosomes from 959 subjects in 20 pedigrees WES data for odd-numbered autosomes from 464 subjects in 20 pedigrees	Longitudinal SBP, real and simulated phenotypes	Support vector machine filter, exclusion of variants with more than 10 % missing calls, extracted with VCFtools
Datta et al. [9]	WES data within *ULK4* and *MAP4* from 1943 unrelated subjects	Cases were defined as persons with a SBP >140 mm Hg, DBP >90 mm Hg or taking antihypertension medication. Other persons, including individuals with a missing medication field, were treated as controls	Exclusion of variants with more than 25 % missing calls or a MAF >0.001, leaving 70 *ULK4* and 18 *MAP4* variants for analysis
Fernández-Rhodes et al. [7]	GWSNPA data for odd-numbered autosomes from 959 subjects in 20 pedigrees	Hypertension phenotype PHEN simulated based on 984 variants with main SBP effects, and 3 CYP3A43 variants that interacted with medication but showed no main effect	Excluded 92 individuals with missing phenotype data; monomorphic and singleton variants were filtered out. Only the last SBP measurement was considered
González-Silos et al. [1]	WES variants in chromosome 3 from 407 samples with information on blood pressure medication out of 1943 unrelated samples	DBP	Reference and alternative allele counts (AD fields in the FORMAT tag of the vcf file), genotype (GT field in the FORMAT tag) and average genotype quality (GQ field in the FORMAT tag), extracted with VCFtools. Nonbiallelic, monomorphic and variants with a MAF <0.003 were excluded, leaving 8957 variants for analysis
Oh [5]	WES data in *MAP4* from 1943 unrelated subjects	Log-transformed baseline measurements of SBP and DBP	Exclusion 92 individuals with missing phenotype data, monomorphic and singleton variants were filtered out
Schwantes-An et al. [6]	WES data in odd-numbered autosomes from 1943 unrelated subjects	Four traits were simulated by the authors under a null hypothesis of no genetic association. The fifth trait was Q1 provided	Alternative allele counts (NALTT field) were extracted with VCFtools and converted to 2-allele genotype calls. Nonbiallelic and monomorphic variants, and variants with more than 5 % missing calls were excluded, leaving 313,340 variants for analysis
Shin et al. [3]	WES data in *MAP4* from 1943 unrelated subjects	Real data: Cases were defined as persons with SBP >140 mm Hg, DBP >90 mm Hg or taking antihypertension medication. Other persons, including individuals with a missing medication field, were treated as controls	Excluded 92 individuals with missing phenotype data
			Predicted alternative allele counts (DOSAGE field) were extracted with VCFtools; monomorphic variants were filtered out, leaving 90 variants for analysis
		Simulated phenotypes: Null trait Q1 (dichotomomized) and PHEN, both with disease prevalence of 17.8 %	
Thompson and Fardo [4]	Variants in *TNN*, *LEPR*, *GSN*, *TCIRG1*, and *FLT3* including 100,000 base pairs upstream and downstream	Simulated phenotypes Q1 and PHEN on 1943 unrelated subjects	Data extracted with VCFtools; monomorphic variants were filtered out
Wang et al. [8]	WES data 5 kb within, up- and downstream of *MAP4* from 1943 unrelated subjects	Simulated data, including a null trait (25 variants have true SBP effects)	Excluded 81 subjects without age information; monomorphic and low-coverage (<20×) variants were filtered out, leaving 94 variants

*DBP* diastolic blood pressure, *GWSNPA* genome-wide single nucleotide polymorphism array, *MAF* minor allele frequency, *NALTT* number of nonreference alleles for each individual thresholded, *SBP* systolic blood pressure, *VCF* variant call format, *WES* whole exome sequence

### New methods for new types of data

The relationship between genetic variability and a given phenotype is usually investigated based on called genotypes. Sequence data provides ancillary information on the distribution of the number of reads at a particular position. This includes the counts of reference and alternative alleles. González Silos et al. hypothesized that allele counts are genotype measurements that are more informative than called genotypes in the sense that the two counts, “no alternative allele out of 100 reads” and “one alternative allele out of 10 reads,” both translate into the same called genotype (reference allele homozygote). In other words, after applying user-defined data quality filters, uncertainty in genotype calling is rarely taken into account in genetic association tests.

To explore association test approaches that rely on allele counts from sequence data as an alternative to called genotypes, González Silos et al. fitted several regression models treating alternative allele counts both as response and as explanatory variables. Negative binomial regression was applied to investigate the relationship between alternative allele counts as response variable, using the total number of reads at a particular position as an offset, and the diastolic blood pressure was adjusted for age, sex, and medication as an explanatory variable. Zero-inflated and Hurdle-negative binomial regression were examined, too, because of their flexibility in the presence of zero inflation. The genotype–phenotype relationship was also investigated based on the ratio “alternative allele count/number of reads”, which was alternatively considered as a response and an explanatory variable in standard and robust linear regression models. Type I error rates were roughly estimated, assuming that most of the investigated variants were under the null hypothesis of no genetic association, and quantile-quantile plots were used to explore possible disparities between small probability values from the investigated regression models. Table 2 lists key concepts addressed in accepted papers from the Population-Based Association group. In addition to allele counts, negative binomial regression models, and extensions thereof, González Silos et al. dealt with the concept of “downsampling.” Table 3 presents related bibliography and publicly available software used by group members.

**Table 2:** Key concepts addressed by authors of accepted papers in the Population-Based Association group

Theme	[Contribution reference] concept
New methods for new data types	[1] Alternative allele count: Number of reads that support a given alternative allele based on individual sequence data
	[1] Negative binomial regression: Type of regression model used to investigate response variables that are counts. In contrast to Poisson regression, negative binomial regression allows for overdispersion—a variance larger than the mean
	[1] Hurdle and zero-inflated models: Two statistical models used to investigate count response variables with a large proportion of zeros. Hurdle models assume that a Bernoulli process determines whether counts are zero or positive. If the response is positive, its conditional distribution is governed by a truncated-at-zero count data model. Zero-inflated models assume the response variable is a mixture of a Bernoulli and a count distribution, eg, negative binomial
	[1] Downsampling: Selecting a subset of the reads in a high-coverage position to improve computational efficiency
Handling rare variants	[2] Variant ascertainment bias: Variant selection criteria, such as minor allele frequency, can influence kinship and population structure estimates
	[2] Kinship estimation: the estimation of relationships among samples based upon genotypes rather than known pedigrees is sensitive to the selected variants and the applied statistical methods
	[2] Population structure: Admixture events leave a signature in the patterns of genetic variation within a population. This can bias genome-wide association studies, and be used as a tool to identify genetic variants influencing a trait
	[3] Firth’s penalized likelihood: A logistic regression likelihood penalized by Jeffrey’s invariant prior. A first-order bias term is introduced into the score function to reduce the bias in the log odds ratio estimate that arises as a result of sparse data
	[3] Small-sample-adjusted score test: A logistic regression score test in which the null distribution of the test statistic is adjusted using estimates of small sample variance and kurtosis
	[3,9] Sequence kernel association test: Variant-collapsing test for a subset of variants constructed by aggregating individual variant score test statistics
	[4] Quantitative trait mapping: The search for positions along the genome associated with quantitative traits
	[4] Tree-based methods: Methods that account for uneven evolutionary relatedness among genetic variants
	[4] Phylogenetic tree: A bifurcating tree used to represent the evolutionary relationships among variants (illustrated in Fig. 2)
	[5] Within-chain permutation: Permutation of individual phenotypes is a widely used strategy to investigate the null distribution. Under the frequentist approach, statistics based on actual data are compared with the distribution of statistics from permuted data sets. In Bayesian analyses, computing time can be reduced by permuting phenotypes within the single Markov chains used to infer posterior distributions.
	[6] Minor allele count (MAC): The total count of minor alleles for all individuals evaluated at a particular position. For rare variants, the MAC reflects better data sparsity than the minor allele frequency
Rare variant behavior	[7] Gene–environment interaction term model: Statistical approach that tests for gene–environment interactions by including a gene–environment interaction term to measure the change in the outcome when both the genetic marker and environmental factor are present, as compared to when one or both factors are not present [7] Environment-stratified models: Alternative approach to identifying gene–environment interactions, by comparing genetic effect sizes between strata defined by an environmental exposure
Follow up of association signals	[8] Bayesian adjustment for confounding: A Bayesian approach for estimating the average causal effect of an exposure on an outcome in observational studies while accounting for the uncertainty in confounder selection. It uses Bayesian model averaging to average inference across many models according to posterior weight determined by a joint model of the exposure and the outcome
	[9] Logistic Bayesian LASSO (least absolute shrinkage and selection operator): Method based on a retrospective likelihood that models the probability of haplotypes given disease status. The odds of disease are expressed as a logistic regression model, whose coefficients are regularized through Bayesian LASSO

**Table 3:** Relevant bibliography and software used by authors of accepted papers in the Population-Based Association group

Topic	Bibliography	Software
New methods for new data types	Satten GA, Johnson HR, Allen AS et al. Testing association without calling genotypes allows for systematic differences in read depth between cases and controls. In: Abstracts from the 22nd Annual Meeting of the International Genetic Epidemiology Society, Chicago IL, USA. ISBN: 978-1-940377-00-1, 2012, 9. Original proposal to use the proportion of calls for the minor allele instead of called genotypes Karazsia BT and Dulmen MHM: Regression models for count data: illustrations using longitudinal predictors of childhood injury. *Journal of Pediatric Psychology* 2008;33:1076–1084. Intuitive examples of widely used models for count data	R-packages stats and pscl to fit negative binomial/linear and zero-inflated/Hurdle-negative regression models, respectively
Handling rare variants	Conomos MP, Miller MB, and Thornton TA. Robust inference of population structure for ancestry prediction and correction of stratification in the presence of relatedness. *Genetic Epidemiology* 2015;39(4):276–293. Reviews the complications of population structure and kinship estimation Kang HM, Sul JH, Service SK, et al. Variance component model to account for sample structure in genome-wide association studies. *Nature Genetics* 2010;42:348–354. Description of EMMAX, an association testing tool for dependent observations	PC-AiR is implemented in R and is available from http://www.bioconductor.org/packages/devel/bioc/html/GENESIS.html
		SNPRelate is an R package, available from http://www.bioconductor.org/packages/release/bioc/html/SNPRelate.html. PC-AiR and SNPRelate were used for kinship estimation.
		EMMAX for genome wide association testing is available from http://genetics.cs.ucla.edu/emmax/
		RFMiX for local ancestry mapping is available from https://sites.google.com/site/rfmixlocalancestryinference/
		R-package pmlr to conduct penalized logistic regression likelihood ratio tests (http://cran.r-project.org/web/packages/pmlr)
		SKAT to perform single-variant score tests, and 3 variant-collapsing tests: burden, nonburden sequence kernel association test, and optimal unified test (http://cran.r-project.org/web/packages/SKAT/)
		Blossoc to estimate phylogenetic trees
	Maples BK, Gravel S, Kenny EE, and Bustamante CD. RFMix: a discriminative modeling approach for rapid and robust local-ancestry inference. *American Journal of Human Genetics* 2013;93:278–288. Description of RFMix, which can be used for local ancestry mapping	R packages ape and geiger to manipulate phylogenetic trees
	Bull SB, Mak C, and Greenwood CMT: A modified score function estimator for multinomial logistic regression in small samples. *Computational Statistics & Data Analysis* 2002;39:57–64	
	Firth D. Bias reduction of maximum likelihood estimates. *Biometrika* 1993;80:27–38	
	Lee S, Emond MJ, Bamshad MJ, et al. Optimal unified approach for rare-variant association testing with application to small-sample case–control whole-exome sequencing studies. *American Journal of Human Genetics* 2012;91:224–237	
	Thompson K, Kubatko L. Using ancestral information to detect and localize quantitative trait loci in genome-wide association studies. *BMC Bioinformatics* 2013;14:200	
	Mailund T, Besenbacher S, and Schierup MH: Whole genome association mapping by incompatibilities and local phylogenies. *BMC Bioinformatics* 2006;7:454	
Rare variant behavior	Tabangin ME, Woo JG, and Martin LJ. The effect of minor allele frequency on the likelihood of obtaining false positives. *BMC Proceedings* 2003;3 Suppl 7:S41	MMAP to fit linear mixed model in a family-based sample, estimate either model-based or robust standard errors, and conduct a 1 df test of gene–environment interactions in an “interaction model” using the estimates gene–environment interaction term
		METAL to estimate 1 df and 2 df tests of gene–environment interactions using a model with a gene–environment interaction term (“interaction model”)
	Goh L and Yap VB. Effects of normalization on quantitative traits in association test. *BMC Bioinformatics* 2009;10:415.	R-package EasyStrata to estimate 1 df and 2 df tests of gene–environment interactions by comparing the genetic effects across environmental strata (“med-diff” approach)
	Manning AK, LaValley M, Liu CT, et al. Meta-analysis of gene-environment interaction: joint estimation of SNP and SNP × environment regression coefficients. *Genetic Epidemiology* 2011;35:11–8. Application of 1° of freedom (df) and 2 df tests of gene–environment interactions using a model with a gene–environment interaction term	
	Randall JC, Winkler TW, Kutalik Z, et al. Sex-stratified genome-wide association studies including 270,000 individuals show sexual dimorphism in genetic loci for anthropometric traits. *PLoS Genetics* 2013;9:e1003500. Application of a 1 df test of gene–environment interactions by comparing the genetic effects across environmental strata	
	Aschard H, Hancock DB, London SJ, and Kraft P. Genome-wide meta-analysis of joint tests for genetic and gene-environment interaction effects. *Human Heredity* 2010;70:292–300. Application of a joint 2 df test of gene–environment interactions and genetic main effects by comparing the genetic effects across environmental strata	
Follow up of association signals	Wang C, Parmigiani G, and Dominici F. Bayesian effect estimation accounting for adjustment uncertainty. *Biometrics* 2012;68:661–671	Codes that implement Bayesian adjustment for confounding are available at http://sweb.uky.edu/~cwa236/
	Wang C, Dominici F, Parmigiani G, Zigler CM. Accounting for uncertainty in confounder and effect modifier selection when estimating average causal effects in generalized linear models. *Biometrics* 2015, in press.	R-packages hapassoc, haplo.stats, LBL to implement the haplotype association methods
	These two papers proposed the Bayesian adjustment for confounding (BAC) method	
	Biswas S and Lin S: Logistic Bayesian LASSO for identifying association with rare haplotypes and application to age-related macular degeneration. *Biometrics* 2012;68:587–597	
	Biswas S, Xia S, and Lin S: Detecting rare haplotype-environment interaction with logistic Bayesian LASSO. *Genetic Epidemiology* 2014;38:31-41	

### Handling rare variants

Blue et al. compared kinship estimators and investigated the ability of principal component analysis to capture ancestry proportions relying on different subsets of sequence data. Kinship was estimated using 4 different approaches (method of moments; maximum likelihood for noninbred pairs; robust Kinship-based INference for Genome-wide association studies; and PC-AiR, a moment estimator that adjusts for population structure using principal components). Three different strategies were applied to select linkage-disequilibrium pruned whole genome and exome sequence variants (agnostic {every 100th variant], selective [variants with a minor allele frequency (MAF) ≥5 %], and homogenizing [variants with similar frequencies in African, Native American, Asian, and European populations]). To examine the ability of principal component analysis to capture ancestry, principal components were estimated relying on a genetic relationship matrix that was calculated based on 4 subsets of whole genome sequence variants: rare (MAF <0.01 or MAF <0.05) and common (MAF >0.01 or MAF >0.05). Table 3 presents and briefly describes recent publications in the field.

The analysis of association between binary phenotypes and single rare variants is challenging because conventional logistic regression approaches often violate the large-sample assumption for test statistics, resulting in poor type I error control and low statistical power. In particular, standard score tests can be extremely anticonservative under the null. Shin et al. explored alternative tests for low-frequency and rare variants, including 2 sparse-data approaches to single-variant tests, and collapsing tests for sets of variants. The first explored sparse-data approach was the Firth-type likelihood ratio test based on the penalized log-likelihood function

$$ {l}_p\left(\boldsymbol{\beta} \right)=l\left(\boldsymbol{\beta} \right)+\frac{1}{2} \log \left(\left|i\left(\boldsymbol{\beta} \right)\right|\right), $$

where *i*(*β*) is the Fisher information matrix. The second sparse-data approach was a modified score test which incorporates small-sample variance and/or kurtosis to obtain the null distribution. The investigated variant-collapsing tests included a MAF-weighted burden test, a nonburden sequence kernel association test (SKAT), and a unified approach that optimally combines SKAT and the burden test (SKAT-O). Investigated variant-collapsing tests were applied to sets of rare variants alone, rare and low-frequency variants, and all variants within defined subregions built according to physical proximity. Table 3 lists the software used (R-package pmlr and SKAT).

**Fig. 2:**
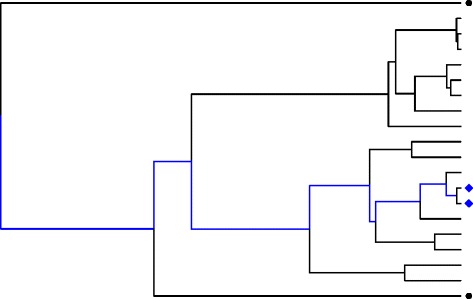
Illustration of the evolutionary history of a particular genetic variant represented by a phylogeny tree. In the phylogenetic tree, time moves from past (left) to present (right). Suppose some of the variants represented in this tree are associated with a trait. Then, a large covariance is expected among trait values from 2 variants (eg, the blue diamonds) sharing a large portion of their evolutionary history (shown by the branches in blue). In contrast, the 2 variants denoted by black circles share a smaller portion of evolutionary history, so that little covariance in the corresponding trait values is expected

Thompson and Fardo compared mapping methods that account for the evolutionary relatedness among individuals (tree-based) with standard methods that ignore evolutionary relationships (non–tree-based association mapping methods). Each genetic variant has an evolutionary history that can be represented by a phylogenetic tree (see Table 2). In Fig. 2, each tip represents a copy of the variant. Time moves from past to present, from left to right, across the tree, and the branch lengths represent time. If 2 variants share a large portion of their evolutionary history (the 2 blue diamonds), associated phenotypic traits are expected to be correlated, whereas if 2 variants share little evolutionary history (illustrated by the 2 black circles), trait values are expected to be uncorrelated. Tree-based association mapping methods that integrate evolution history in the statistical analysis may have improved ability to detect weaker associations but existing tree-based methods are unable to consider external covariate information and are computationally expensive. Related literature and software in the field is sparse (see Table 3).

The identification of rare-variant associations by multimarker approaches, for example, collapsing, simple-sum, and weighted-sum methods, has recently drawn much attention. Table 3 presents a noninclusive list of available software. These methods first collapse rare variants and then implement a LASSO (least absolute shrinkage and selection operator), partial least-squares regression model, or other supporting statistical method that relies on common and collapsed rare variants. Variant pooling may dilute rare variant effects, and the cutoff definition to distinguishing rare from common variants is arbitrary. To circumvent these limitations, Oh extended previous work and presented a Bayesian variable selection approach to detect associations with both rare and common genetic variants. Under his approach, rare variant mapping is framed as a variable selection problem in a sparse space where risk index scores are constructed for a group of rare variants over the genomic region. Technical details on the chosen priors and on inference relying on marginal posterior probabilities of latent variables can be found in the GAW 19 proceedings. Table 2 provides a brief explanation of within-chain permutation of phenotype data to calculate empirical thresholds and adjust false-positive rates.

### Rare-variant behavior

In next-generation sequence data, the proportion of rare variants is substantially larger than the proportion of common variants typically measured in array-based genome-wide association studies. Rare variants present a challenge because often there are too few of the rare alleles for traditional statistical tests, and the increased variant density results in multicollinearity, making it difficult to identify independent associations. Schwantes-An et al. investigated the effect of the MAF, chromosomal position, significance threshold, and departure from normality of the investigated phenotype on the type I error rate. Five quantitative phenotypes were simulated under the null hypothesis. The first phenotype followed a standard normal distribution, the second followed a gamma distribution. The third and fourth phenotypes were the log_10_ (rank-based inverse normal) transformations of the second phenotype. The fifth phenotype was the null trait Q1 provided by workshop organizers.

Fernández-Rhodes et al. compared type I error rates and statistical power for 2 gene–environment interaction methods. The first method (“interaction model”) uses a gene–environment interaction term to measure the change in outcome when both the genetic marker and environmental factor are present, compared to the presence of the genetic marker alone. The second method tests for effect-size differences between strata under distinct environmental exposures (medication vs nonmedication in the present context, “med-diff” approach). The 2 methods can be applied to test gene–environment interactions with 1° of freedom (df), as well as to test both main genetic and gene–environment interaction effects with 2 df tests. They were compared relying on genotype-medication interactions simulated by the GAW19 organizers. Gene–environment interaction analyses were adjusted for age, sex, population structure, and family relatedness.

### Follow up of association signals

Estimation of causal effects of genetic variants on disease may help to bridge the gap between assessment of association and function, allowing at the same time an improved localization of disease variants. The causal effect of genetic variants on clinical phenotypes may be confounded by demographic and clinical characteristics, and also by other genetic variants as a result of linkage disequilibrium. Wang et al. explored the estimation of the average causal effect of genetic variants on clinical phenotypes using the Bayesian adjustment for confounding method. This method has been proposed to estimate average causal effects in the presence of many confounders, assuming all of them have been measured.

Bayesian adjustment for confounding utilizes a Bayesian model averaging approach and estimates causal effects by a posterior weighted average of average causal effect estimates from a battery of models with different sets of covariates. In contrast to standard Bayesian model averaging, Bayesian adjustment for confounding incorporates the strength of associations between covariates in the model and the exposure into the prior. It has been shown that the method tends to give large posterior weights to models that have been fully adjusted for confounding, thus resulting in unbiased causal effect estimates (see articles by Wang et al. listed in Table 3). The bias and variability of average causal effect estimates were examined by comparison with a “true model” (age, sex, their interaction, and 25 variants with true systolic blood pressure [SBP] effects), and a “full model” (age, sex, their interaction, smoking status, and 94 variants within, up-, and downstream of *MAP4*). Table 3 provides a link to software developed by Wang et al.

Haplotype analysis is a typical follow-up step after identification of single-association signals. Haplotype-based methods can be more powerful than single–single nucleotide polymorphism (SNP) methods when causal variants are not genotyped, and when multiple variants act in *cis*. In some situations, they also have increased power to detect rare-variant associations over recently developed “collapsing” methods. Datta et al. investigated possible associations with rare haplotypes in 2 hypertension-associated genes, *ULK4* and *MAP4*. They analyzed sliding haplotype windows of 5 variants using 4 haplotype association methods: haplo.score, haplo.glm, hapassoc, and logistic Bayesian LASSO (LBL) and the three collapsing methods (SKAT, SKAT-O and SKAT-RC, see Tables 2 and 3). LBL models the probability of haplotypes given disease status. Unobserved haplotypes are treated as missing data and the frequencies of haplotype pairs are modeled, allowing for Hardy-Weinberg disequilibrium. The odds of disease are expressed as a logistic regression model, whose coefficients are regularized through a double-exponential prior centered at 0 and a variance parameter, which is further assigned a hyper prior. By regularizing regression coefficients through their prior distributions, the LBL weeds out unassociated (especially common) haplotypes, allowing the associated rare haplotypes to be detected more easily.

## Results and discussion

**Fig. 3:**
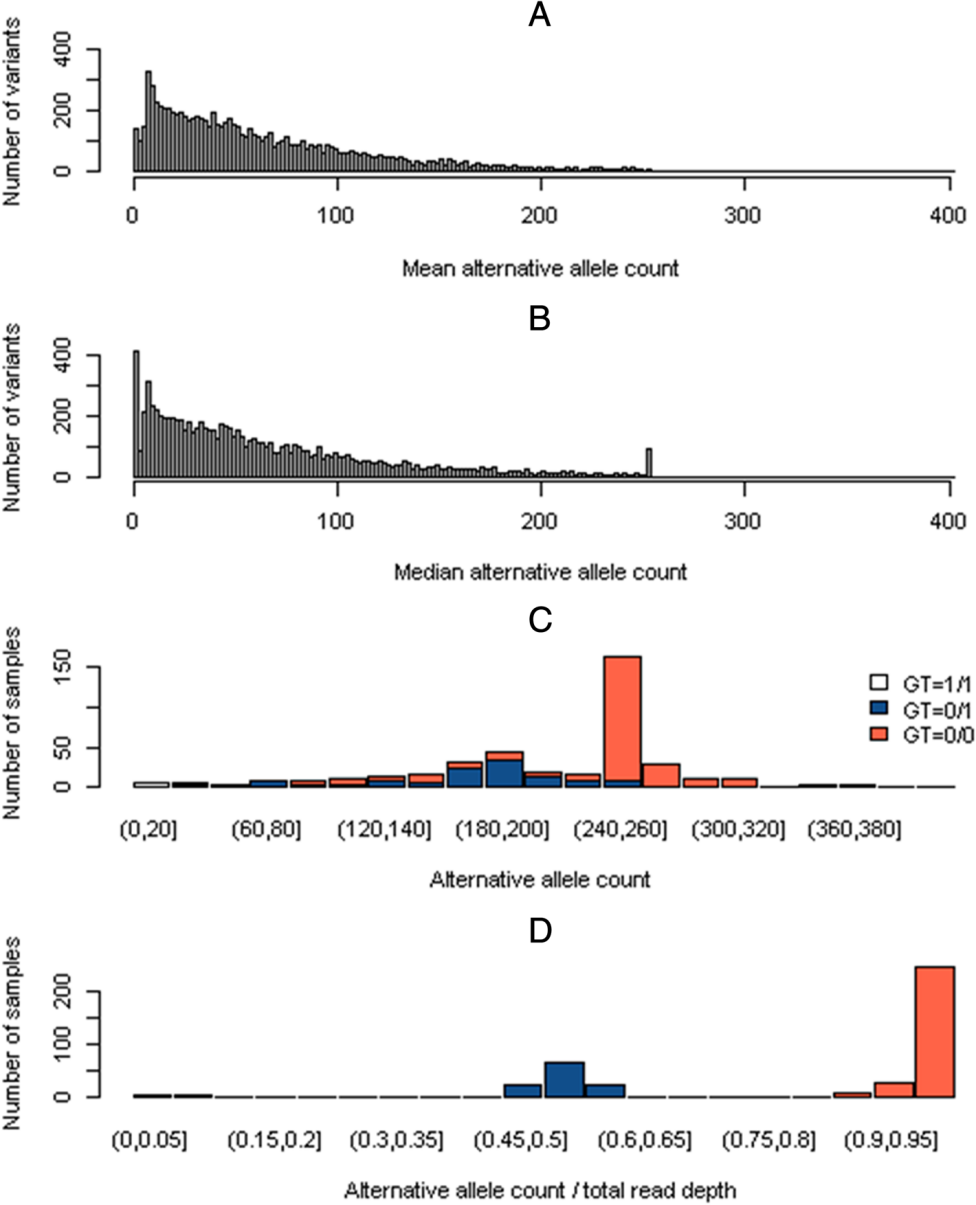
Distribution of alternative allele counts. Mean alternative allele counts (AACs) per variant (**a**), median AACs per variant (**b**), exemplary AAC distribution grouped by genotype for the variant in position Chr3:16249998 with minor allele frequency equal to 0.17 (**c**), and exemplary comparison of the ratios (AAC/total read depth) and called genotypes for the variant in position Chr3:16249998 (**d**)

Figure 3 shows the distribution of alternative allele counts for the investigated variants in chromosome 3 as an alternative to called genotypes. In contrast to the histogram of mean counts *(Panel A)*, the histogram of median counts revealed that most variants had a median count of zero *(Panel B)*. González Silos et al. found 105 variants with a median count exactly equal to 254 *(Panel B)*. The origin of this peak was investigated but, unfortunately, it could not be unveiled and information on downsampling was not available. *Panel C* represents a histogram of alternative allele counts for the variant in position Ch3:16249998, which presented a median (mean) count of 254 (40.03). This variant showed a MAF of 0.168 (280 reference allele homozygotes, 117 heterozygotes, and 10 alternative allele homozygotes). *Panel D* compares the distributions of the ratio “alternative allele count/number of reads” and called genotypes.

The investigated regression models with the best control of type I error rates were zero-inflated and Hurdle-negative binomial regression for the relationship between alternative allele counts and adjusted diastolic blood pressure, and standard and robust linear regression for the relationship between the ratio “alternative allele count/number of reads” as response variable and adjusted diastolic blood pressure as explanatory variable. The simultaneous consideration of ability to discriminate variants with small associated probability values, occurrence of convergence problems, and robustness of probability values against departing blood pressure observations indicated that Hurdle-negative binomial regression constitutes a promising approach.

Regarding the selection of variants for kinship estimation, markers that were not ancestry informative resulted in the most accurate estimates. The homogenizing selection strategy with the maximum likelihood and PC-Relate estimators assigned correct relationships for more than 90 % of pairs of first- and second-degree relatives and unrelated subjects. All methods assigned relationships incorrectly for 20 % of third- and fourth-degree relative pairs. European and Native American ancestries were best captured by principal component analysis based on common variants, with similar results for variants with a MAF >0.01 or >0.05. The ability to capture African ancestry was poorer, and resulted as maximized when principal components relied on variants with a MAF <0.05. Clearly allele frequency plays an important role in estimates of relatedness and population structure, and should be carefully considered in such analyses.

Concerning the alternative tests for low-frequency and rare variants investigated by Shin et al., the penalized logistic likelihood ratio test and the small-sample modified score test were both better choices than standard single-variant tests. In tests of association between a simulated binary hypertension phenotype and variants in the *MAP4* gene, the sparse-data methods generally improved the control of type I errors. Statistical power was sufficient to detect low-frequency and common variants, but remained low for the rare variants. The occurrence of conflicting *p values* from standard and small-sample tests of the same hypothesis can indicate that large-sample approximations are invalid. Although previous studies have found variant-collapsing tests to have higher power than single-variant tests for rare variants, simulation results for *MAP4* showed low power for variant-collapsing tests when they only included rare variants. The statistical power increased when low-frequency variants were included. Because the power of variant-collapsing tests depends on the number, frequency, and effect sizes of associated and neutral variants, identification of better grouping and weighting strategies may translate into an improved power to detect regions that only contain rare variants.

With reference to the consideration of evolutionary relationships, the type I error rate of tree-based mapping methods appeared to be well controlled, whereas non–tree-based association mapping showed inflated type I error rate for all 5 investigated genes. Regarding detection, both methods performed well when analyzing genes with large-effect variants, and both methods showed small power in the analysis of genes with low-penetrance variants. Regarding localization of true causal loci, both methods showed similar mapping abilities for genes with large effects. In the case of small-effect variants, tree-based outperformed non–tree-based association mapping, which may point to an advantage of using evolutionary information to detect weak genetic signals.

Under the Bayesian variable selection approach to detect associations with both rare and common genetic variants, marginal posterior probabilities were quite robust against the MAFs used to define variant rarity (from 0.05 to 5 %), and they clearly surpassed empirical probabilities based on permuted phenotypes for several positions, pointing to associations between rare and common variants in *MAP4* with SBP and diastolic blood pressure (DBP).

In agreement with Fig. 3 from González Silos et al., 77 % of the variants investigated by Schwantes-An et al. were extremely rare (MAF <0.0025), and more than half had the variant allele in only a single individual. Type I error rates were not inflated for the normal and rank-based inverse normal transformed gamma null phenotypes. However, type I error rates for the gamma and log-transformed gamma-distributed phenotypes increased with decreasing MAFs, with increasing departure from normality, and with decreasing significance thresholds. Although Q1 was nearly normally distributed, type I error rates for common variants were higher than expected. The inflation of type I error rates for rare and extremely rare variants for null traits that departed from normality was ameliorated by transforming nonnormally distributed traits to those with a more normal distribution.

Regarding the behavior of rare variants interacting with environmental factors, type I error rates did not surpass the expected nominal 5 % significance threshold for either of the 2 investigated gene–environment interaction methods (“interaction model” and “med-diff” approach to test effect size differences between strata) for variants with at least a MAF of 1 %. The statistical power was higher for the “med-diff” approach for variants with a MAF <1 %, but it was higher for the “interaction model” when a variant with a MAF >5 % was evaluated. Nonconvergence was a limitation of the “med-diff” approach.

Finally, in respect of the follow up of association signals, mean squared errors were smaller for estimates based on Bayesian adjustment for confounding than for full-model–based estimates of the average causal effect in the investigated scenarios. The reduced variation of estimated average causal effects was a result of the simultaneous consideration of an exposure model and an outcome model in the Bayesian adjustment for confounding, suggesting that this method is able to efficiently estimate the causal effect of genetic variants.

Rare-variant haplotype analyses revealed that “hapassoc” often showed convergence problems and, when it converged, association results were similar to that of “haplo.glm.” The haplotypes found to be associated depended on the method. The ranking of methods by the total number of significant haplotypes found on the 2 genes was LBL < haplo.score < haplo.glm. However after permutation of the case–control status to mimic the null scenario, the ratio of associated haplotypes to the total number of haplotypes was also lower for LBL than for “haplo.score” and “haplo.glm,” indicating a controlled false-positive rate for LBL compared to “haplo.score” and “haplo.glm.” SKAT and its variants did not identify statistically significant association signals. Based on these results, haplotype association methods seem to be useful and complementary to collapsing approaches for sequence data.

## Conclusions

With their results, members of the Population-Based Association group identified several current methodological gaps regarding both the preparation and the statistical analysis of sequence data. Sequence data is noisy, and the investigation of the distribution of allele counts instead of relying on called genotypes could offer some advantage. The selection of genetic variants was found to play a major role in the assessment of population structure and cryptic relatedness. Most statistical methods with good properties for common variants were found inappropriate for rare ones. Methodological gaps were also identified in the follow up of association signals. Novel methods are needed to investigate rare haplotypes and interactions between the environment and rare variants found in sequence data, as well as for causal effect estimation.

## References

[1] González Silos R, Karadag Ö, Peil B, Fischer C, Kabisch M, Legrand C (2015). Using next generation DNA sequence data for genetic association tests based on allele counts with and without consideration of zero-inflation. BMC Proc.

[2] Blue EM, Brown LA, Conomos MP, Kirk JL, Nato AQ, Popejoy AB (2015). Estimating relationships between phenotypes and subjects drawn from admixed families. BMC Proc.

[3] Shin JH, Yi R, Bull SB (2015). Identification of low frequency and rare variants for hypertension using sparse-data methods. BMC Proc.

[4] Thompson KL, Fardo DW (2015). Comparing performance of non-tree based and tree-based association mapping methods. BMC Proc.

[5] Oh C (2015). Identifying rare and common variants with Bayesian variable selection. BMC Proc.

[6] Schwantes-An TH, Sung H, Sabourin JA, Justice CM, Sorant AJM, Wilson AF (2015). Type I error rates of rare single nucleotide variants are inflated in tests of association with non-normally distributed traits using standard linear regression methods. BMC Proc.

[7] Fernández-Rhodes L, Hodonsky CJ, Graff M, Love SM, Howard AG, Seyerle AA (2015). Comparison of two models for gene-environment interactions: an example of simulated gene-medication interactions on systolic blood pressure in family-based data. BMC Proc.

[8] Wang C, Liu J, Fardo DW (2015). Causal effect estimation in sequencing studies: a Bayesian method to account for confounder adjustment uncertainty. BMC Proc.

[9] Datta A, Zhang Y, Zhang L, Biswas S (2015). Association of rare haplotypes on *ULK4* and *MAP4* genes with hypertension. BMC Proc.

